# Predictors of healthy physiological aging across generations in a 30-year population-based cohort study: the Doetinchem Cohort Study

**DOI:** 10.1186/s12877-023-03789-2

**Published:** 2023-02-23

**Authors:** Bette Loef, Gerrie-Cor M. Herber, Albert Wong, Nicole A. H. Janssen, Jurriaan Hoekstra, H. Susan J. Picavet, W. M. Monique Verschuren

**Affiliations:** 1 Center for Nutrition, Prevention and Health Services, National Institute for Public Health and the Environment, Bilthoven, The Netherlands; 2 Julius Center for Health Sciences and Primary Care, University Medical Center Utrecht, Utrecht University, Utrecht, The Netherlands

**Keywords:** Exposome, Longitudinal study, Physiological aging, Prediction

## Abstract

**Background:**

Predicting healthy physiological aging is of major interest within public health research. However, longitudinal studies into predictors of healthy physiological aging that include numerous exposures from different domains (i.e. the exposome) are scarce. Our aim is to identify the most important exposome-related predictors of healthy physiological aging over the life course and across generations.

**Methods:**

Data were used from 2815 participants from four generations (generation 1960s/1950s/1940s/1930s aged respectively 20–29/30–39/40–49/50–59 years old at baseline, wave 1) of the Doetinchem Cohort Study who were measured every 5 years for 30 years. The Healthy Aging Index, a physiological aging index consisting of blood pressure, glucose, creatinine, lung function, and cognitive functioning, was measured at age 46–85 years (wave 6). The average exposure and trend of exposure over time of demographic, lifestyle, environmental, and biological exposures were included, resulting in 86 exposures. Random forest was used to identify important predictors.

**Results:**

The most important predictors of healthy physiological aging were overweight-related (BMI, waist circumference, waist/hip ratio) and cholesterol-related (using cholesterol lowering medication, HDL and total cholesterol) measures. Diet and educational level also ranked in the top of important exposures. No substantial differences were observed in the predictors of healthy physiological aging across generations. The final prediction model’s performance was modest with an R^2^ of 17%.

**Conclusions:**

Taken together, our findings suggest that longitudinal cardiometabolic exposures (i.e. overweight- and cholesterol-related measures) are most important in predicting healthy physiological aging. This finding was similar across generations. More work is needed to confirm our findings in other study populations.

**Supplementary Information:**

The online version contains supplementary material available at 10.1186/s12877-023-03789-2.

## Introduction

Population aging is a global phenomenon that will continue to affect all regions of the world [1]. As people live longer, spending these later years in good health and well-being is becoming increasingly important for present and future generations. Therefore, healthy aging, defined by the World Health Organization (WHO) as a process of developing and maintaining the functional ability that enables well-being in older age [1], is a major area of interest within public health research.

Identifying predictors of healthy physiological aging may help to (early) detect people who are (un)likely to remain healthy throughout their life course. Furthermore, insight in these predictors can support health professionals in establishing a more tailored approach to advice individuals who are at increased risk for unhealthy physiological aging. Healthy physiological aging can be influenced by a multitude of factors to which people are exposed over the life course. Therefore, to identify predictors of healthy physiological aging, an ‘exposome approach’ is advocated, taking into account a broad range of exposures from different domains (i.e. specific/general external, and internal environment) [2].

Although prediction research into healthy aging is scarce, previous risk assessment studies found a healthy lifestyle to be related to healthy aging [3–9]. Socio-demographic characteristics [6, 10, 11], health-related exposures (e.g. health conditions) [6, 11], and psychosocial exposures [8, 11] have also been found to be associated with a high likelihood of healthy aging. Risk factors of healthy aging identified in these studies can be useful in *prediction* research. However, most of these previous risk assessment studies included a small number of exposures [3–5, 7–10]. And the two studies that did focus on a broad range of exposures did not provide a direct comparison of the relative importance of predictors of healthy aging [6, 11]. It is therefore unclear what exposures are the most important predictors of (but are not necessarily causally related to) healthy aging. Furthermore, previous studies were either cross-sectional [3, 8, 10] or did not take possible changes of exposures over the life course into account [4, 5, 7, 11]. In addition, younger and older generations may differ in the extent to which they are exposed to a variety of factors [12] and generations may also differ in the strength of the associations of these factors with healthy aging. Therefore, it is possible that predictors of healthy aging differ across generations. However, this area has been under-researched.

In view of these considerations, we aim to identify exposures that were repeatedly measured over the life course that are the most important predictors of healthy physiological aging. We also aim to study whether predictors of healthy physiological aging differ across generations. As a measure of healthy physiological aging, we use the Healthy Aging Index (HAI), based on previous work of Sanders et al. (2012) [13] and Dieteren et al. (2020) [14]. The HAI consists of five indicators, i.e. blood pressure, glucose, creatinine, lung function, and cognitive functioning, representing five major physiological systems (i.e. cardiovascular system, glucose metabolism, kidneys, lungs, and brain) [13], and is shown to be a valid predictor of mortality, morbidity, and disability [13, 15–17]. The focus of the World Health Organization’s definition of healthy aging is on ‘functional ability’, which is the result of the interaction of all physical and mental capacities that a person can draw on (i.e. one’s intrinsic capacity) with a person’s environment [1]. As the HAI used in the current study can be seen as an indicator of intrinsic capacity (for a number of organ systems), our focus is on *physiological* healthy aging. For the purpose of the current study, we apply our recently proposed approach, in which machine learning is used to identify longitudinal exposome-related predictors of health in a population-based study of adults with repeated measurements over 30 years [18].

## Methods

### Study design and population

In the current study, data were used of the Doetinchem Cohort Study [19, 20]. This is a population-based prospective study that studies the impact of (changes in) lifestyle factors and biological risk factors on various aspects of health and well-being of Dutch adults living in Doetinchem. It currently consists of six repeated measures with 5-year intervals over a 30-year period. In 1987–1991, self-completed questionnaires were collected and a physical examination was performed on a random sample of 12,404 (response rate: 62%) participants aged 20–59 years from the town of Doetinchem. Of those, a random sample of 7768 was re-invited to be examined in 1993–1997 (wave 2, *n* = 6,113) and again in 1998–2002 (wave 3, *n* = 4,916), 2003–2007 (wave 4, *n* = 4,520), 2008–2012 (wave 5, *n* = 4,017), and 2013–2017 (wave 6, *n* = 3,437). Response rates varied from 75 to 79%. For the current study, 2,815 participants with complete data on the outcome in this study (i.e. the HAI) at wave 6 were included. Measurements of the exposures were based on data from wave 1 through 5.

### Outcome

The outcome measure of the current study is the age-adjusted HAI, measured at wave 6 when participants were between 46–85 years old. On each of the five indicators of the HAI (i.e. blood pressure, glucose, creatinine, lung function, and cognitive functioning), participants can score 0, 1, or 2 points, where 0 is the least healthy outcome, 1 is the intermediate outcome, and 2 is the healthiest outcome [13, 14]. Table 1 shows the cut-off points that were used to score systolic blood pressure (average score of two measurements), random blood glucose (measured in a peripheral blood plasma sample), creatinine (measured in a peripheral blood plasma sample), forced vital capacity (measured by trained paramedics using a heated pneumotachometer), and cognition (measured by four neuropsychological tests that measure global cognitive function, memory, information processing speed, and cognitive flexibility. Summary score was transformed into a z-score to capture decline rate over time) [14]. These cut-off points were based on previous studies (for blood pressure, creatinine, and forced vital capacity) or based on clinical cut-off points (for blood glucose and cognition) [14]. The total HAI score was calculated by taking the sum of the score of all five indicators, with a range from 0 to 10, with 10 being the healthiest score [14]. Because age is a major predictor of healthy physiological aging and in the current study we were interested in other predictors than age, the age-adjusted HAI was used as an outcome measure. To this end, the average HAI score for each age was subtracted from the HAI score of an individual of that particular age. The resulting score provides an indication as to how an individual fares when compared to his/her age peers. More specifically, a score of 0 indicates an average HAI score for the participant’s age, a negative score indicates a relatively poor HAI score for the participant’s age, and a positive score indicates a relatively good HAI score for the participant’s age.

**Table 1:** Cut-off points used to categorize each indicator of the Healthy Aging Index (adapted from Dieteren et al. 2020)

		**Score**		
**Healthy Aging Index indicators**		*0* = *least healthy*	*1* = *intermediate*	*2* = *healthiest*
Systolic blood pressure (mmHg)		≥ 143	126–143	< 126
Random blood glucose (mmol/L)		≥ 11.1	5.6–11.1	< 5.6
Creatinine (mmol/L)	Men	≥ 114.9	97.2–114.9	< 97.2
	Women	≥ 88.4	70.7–88.4	< 70.7
Forced vital capacity (L)	Men	< 3.2	3.2–3.8	≥ 3.8
	Women	< 2.1	2.1–2.6	≥ 2.6
Cognition (Z-score)		< 10th percentile	10th percentile-0	> 0

### Exposures

The exposures included in the current study were divided into four domains. These domains correspond with the specific external environment (lifestyle exposures), the general external environment (environmental exposures), and the internal environment (biological exposures) of the exposome concept [2]. In addition, demographic exposures were taken into account. All included exposures are described in Table S 1 (including in which waves they were measured), and shortly described below:

- *Demographic exposures* consisted of the following: sex (men; women), educational level based on highest level of education attained (primary education or less; lower vocational education or lower secondary education; intermediate vocational education or higher secondary education; higher vocational education or university), nationality (born in the Netherlands; born elsewhere), marital status (single; married; widowed; divorced), household composition (living with partner; living with partner and children; single-parent household; single household; other household), working hours (in hours per week).
- *Lifestyle exposures* were measured by questionnaire, and consisted of the following: alcohol use (never; former; current user, and amount among users), smoking (never; former; current smoker, current amount among users, and packyears), physical activity (occupational physical activity and time spent on moderate to vigorous physical activity per week measured using the EPIC Physical Activity Questionnaire [21]), and sleep (≤ 5; 6; 7; 8; ≥ 9 h per day). Diet was measured using a self-administered Food Frequency Questionnaire based on which the Dutch Healthy Diet index 2015 (DHD15) was calculated, a measure of diet quality, which estimates the level of adherence to the Dutch dietary guidelines [22].
- *Environmental exposures* consisted of exposures in the physical (outside and inside the participants’ home) and social environment. Air pollution (NO_2_, PM_2.5_, and elemental carbon concentration), noise (rail and road traffic noise levels), and green space (normalized difference vegetation index (NDVI) in buffer of 300 and 1000 m) at the participants’ home addresses were measured using dispersion models [23], Standard Model Instrumentation for Noise Assessments [24], and the NDVI derived from Landsat 5 Thematic Mapper data [25, 26]. The presence of damp stains, mold growth, and pets in the house, the hot water supply in the house, the heat source for cooking, and smoking in the participant's environment was self-reported. The social environment was assessed by presence of social support (positive and negative social experiences [27], social support for elderly [28]) and loneliness [29].
- *Biological exposures* consisted of anthropometric exposures measured by trained staff according to standardized protocols (body mass index (BMI), waist circumference, waist/hip ratio), exposures measured in blood (total and HDL cholesterol), and self-reported use of cholesterol lowering medication. To calculate BMI, body weight and height were measured by trained staff with participants wearing light indoor clothing without shoes, with emptied pockets.

### Statistical analysis

To study which exposures had the greatest predictive value for healthy physiological aging, we followed the statistical analysis steps as described previously in our tutorial paper [18]. These steps were conducted for the total study population (*n* = 2,815) and for each of the four generations (i.e. generation 1960s/1950s/1940s/1930s) separately. Participants in generation 1960s were aged 20–29 years at baseline and on average 53 years at wave 6 (*n* = 459), those in generation 1950s were aged 30–39 years at baseline and on average 60 years at wave 6 (*n* = 1155), those in generation 1940s were aged 40–49 years at baseline and on average 70 years at wave 6 (*n* = 872), and those in generation 1930s were aged 50–59 years at baseline and on average 79 years at wave 6 (*n* = 329). Analyzes were performed using R Version 4.0.2. (http://www.R-project.org/).

First, we determined the average level of exposure over the life course and the average trend in exposure over the life course. To this end, all exposures that were measured at multiple waves of the study were summarized in the average of the exposure at wave 1 through 5 (called the Area-Under-the-Exposure, AUE) and the average trend in the exposure over time (called the Trend-Of-the-Exposure, TOE) (for further details see Text S1 and [18]).

Secondly, random forest (RF) [30] was used to analyze which longitudinal exposures best predicted the HAI (R-package randomForest [31]). RF is a non-parametric machine learning algorithm that consists of an ensemble of decision trees that predict the outcome measure. To determine the prediction performance of the RF algorithm, the root mean square error (RMSE), explained variance (R^2^), and mean-absolute error (MAE) were used. Parameters of the RF algorithm (i.e. size of random sample of exposures used at each split (mtry), number of trees (ntree), minimum number of observations in the final nodes (nodesize), and maximum number of terminal nodes (maxnodes)) were tuned to improve prediction performance [32, 33]. To this end, the dataset was divided in a random 80% training and 20% test dataset. Next, the combination of settings of the tuning parameters that produced the highest prediction performance on the training dataset was selected using a grid search in combination with fivefold cross-validation with R-package caret [34]. The model with the optimal settings was used to make predictions on the test dataset and the corresponding RMSE, R^2^, and MAE were determined and compared with a model without any exposures that simply predicts the training dataset mean (i.e. the null model).

Thirdly, using the optimal parameter settings and fitting a RF on the entire dataset, the variable importance ranking was determined. In this list, the most important exposures that predict the HAI are ranked based on the percentage increase in the mean square error (MSE) when a particular exposure is permuted randomly from the RF model with all exposures.

Fourthly, a post-hoc cross-validation procedure was conducted in which the relation between a x number of top-ranked exposures and the prediction performance was evaluated. Afterwards, the RMSE was estimated for each choice of x, and plotted against each other. The optimal value for x was chosen based on the flattening of the resulting curve. Subsequently, the top-ranked predictors were listed for the total study population and for each generation separately. We additionally calculated the average ranking of the top-ranked predictors of the four generations together.

Lastly, for the most important exposures selected through cross-validation, the relation between the exposure and the HAI was visualized using partial dependence plots (PDP) [35]. PDPs plot the value of the average predicted outcome on the y-axis against each value of the exposure on the x-axis, while keeping all other exposures constant at their original values.

## Results

Table 2 presents the characteristics of the total study population and of the four generations for a selection of the 86 included demographic, lifestyle, environmental, and biological exposures (see Table S 2 for an overview of all exposures) and the HAI. In the total study population (*n* = 2,815), 53.3% of the participants were women, participants were on average 49.4 years (SD = 8.4), and 26.7% of the participants had completed higher vocational education or university (Table 2). The mean score on the crude HAI scale (from 0–10, higher score indicates better health) was 7.2 (SD = 1.8) in the total study population at wave 6. The age-adjusted HAI score ranged between -6.6 and 4.9, with a mean score of 0 (SD = 1.6).

**Table 2:** Characteristics of the study population for a selection of the included exposures and the Healthy Aging Index

Exposure/outcome	Type	Label	Total (*n* = 2815)		Generation 1960s (*n* = 459)		Generation 1950s(*n* = 1155)		Generation 1940s (*n* = 872)		Generation 1930s (*n* = 329)	
			*Mean/%*	*SD/n*	*Mean/%*	*SD/n*	*Mean/%*	*SD/n*	*Mean/%*	*SD/n*	*Mean/%*	*SD/n*
*Demographic exposures*												
Sex	%	women	53.3	1501	59.3	272	52.5	606	53.0	462	48.9	161
Age^a^	AUE	in years	49.4	8.4	37.9	1.7	45.6	3.0	54.7	2.8	64.5	2.7
Age^a^	TOE	in years	5.2	0.1	5.2	0.1	5.2	0.1	5.2	0.1	5.2	0.1
Educational level	%	higher vocational education or university	26.7	752	22.4	103	30.9	357	24.2	211	24.6	81
*Lifestyle exposures*												
Smoking pack years	AUE	in pack years	8.8	11.5	5.2	7.0	8.4	10.3	9.9	12.8	12.0	15.2
Smoking pack years	TOE	in pack years	0.8	2.2	0.9	1.7	1.0	2.2	0.6	2.1	0.6	2.5
Dutch Healthy Diet index 2015	AUE	on a scale from 0 – 130	66.1	11.8	63.0	11.2	65.7	11.7	67.2	11.8	69.3	12.0
Dutch Healthy Diet index 2015	TOE	on a scale from 0 – 130	1.2	6.5	0.8	6.7	1.0	6.4	1.3	6.4	1.6	6.4
*Environmental exposures*												
NO_2_ concentration	AUE	in ug/m^3^	27.7	1.8	27.5	1.7	27.8	1.7	27.7	2.0	28.0	2.1
NO_2_ concentration	TOE	in ug/m^3^	-1.6	0.6	-1.6	0.7	-1.6	0.6	-1.6	0.6	-1.5	0.5
Smoking in participant's environment	% of the time	yes, at home and/or at work	45.2	44.4	49.0	43.8	47.7	44.8	45.5	44.6	31.7	40.6
Smoking in participant's environment	%	from no to yes	6.0	153	7.0	28	7.2	74	4.7	38	4.1	13
*Biological exposures*												
Body mass index	AUE	in kg/m^2^	25.6	3.5	24.6	3.3	25.4	3.4	26.0	3.4	26.6	3.5
Body mass index	TOE	in kg/m^2^	0.6	0.7	0.8	0.8	0.7	0.7	0.5	0.7	0.4	0.6
Use of cholesterol lowering medication	% of the time	yes	5.3	14.2	1.0	5.5	3.3	10.8	7.4	16.0	12.9	21.9
Use of cholesterol lowering medication	%	from no to yes	12.2	342	2.8	13	9.4	108	16.7	146	22.8	75
*Outcome*												
Healthy Aging Index (crude)	at wave 6	on a scale from 0 – 10	7.2	1.8	8.2	1.3	7.7	1.5	6.7	1.8	5.5	1.8
Age-adjusted Healthy Aging Index	at wave 6	on a scale from -10 – 10	0.0	1.6	0.0	1.3	0.0	1.5	0.0	1.7	0.0	1.8

*AUE* Area-Under-the-Exposure, *TOE* Trend-Of-the-Exposure

^a^Age is shown here for descriptive purposes only, as age is not included as an exposure in the analyses, and only used to create the age-adjusted Healthy Aging Index outcome

### Predictors of healthy physiological aging in the total study population

RF was used to examine which longitudinal exposures best predicted the HAI at wave 6 in the total study population. After tuning the RF parameters in the training dataset, the optimal RF model was fitted on the test dataset. In this model, the RMSE (1.43 vs. 1.58), R^2^ (17.40% vs. 0.00%), and MAE (1.14 vs. 1.27) were modestly improved compared to the null model.

**Fig. 1:**
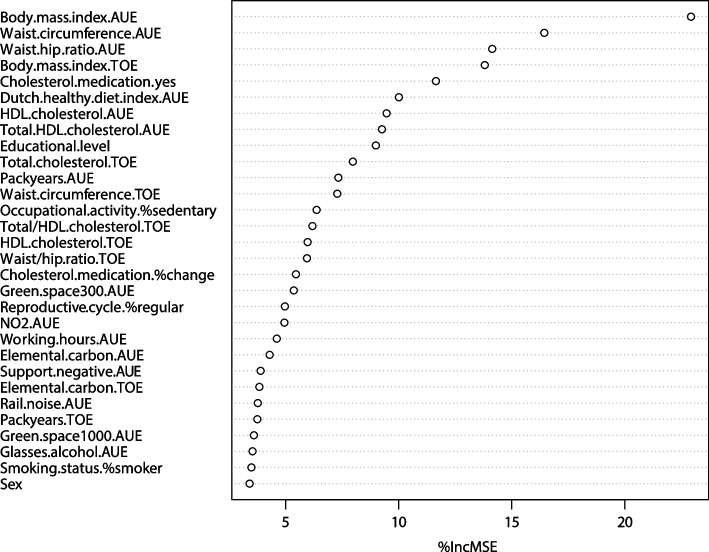
Variable importance ranking of the 30 most important exposures in predicting the Healthy Aging Index. The x-axis displays the percentage increase in the mean square error (MSE) that occurs when a particular exposure is permuted randomly in the random forest. AUE, Area-Under-the-Exposure; TOE, Trend-Of-the-Exposure

**Fig. 2:**
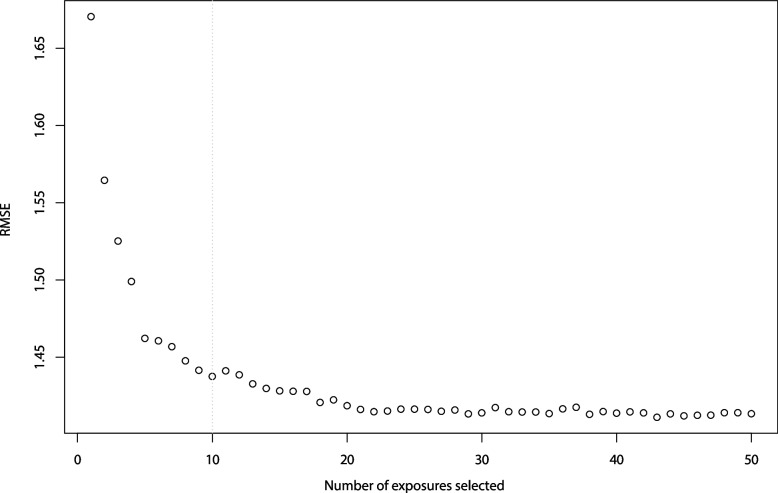
Exposure selection through cross-validation showing the prediction performance (root mean square error, RMSE) (y-axis) of the model using a particular number of top-ranked exposures (x-axis). The dotted gray line represents the optimum number of exposures to select (x = 10)

Figure 1 presents the 30 top-ranked exposures in predicting the HAI based on the RF model with the optimal settings performed on the entire dataset. Next, the number of top-ranked exposures needed to obtain an approximately equally good prediction performance as in the full model with all 86 exposures was determined by stepwise inclusion of the top-ranked exposures in a cross-validation procedure on the training dataset (Fig. 2). The sharpest decrease in the RMSE was seen after selecting the first five exposures (RMSE = 1.46) and slightly further decreased when selecting 6 to 10 exposures (RMSE = 1.44). As the curve started to flatten after 10 exposures, the RMSE slightly increased after 11 exposures, and the RMSE only slightly further decreased when selecting more exposures, the optimum number of top-ranked exposures was set at 10 exposures. The quality metrics of the model with only the 10 top-ranked exposures were virtually the same as the metrics of the model with all 86 exposures (RMSE: 1.44 vs. 1.43, R^2^: 17.00% vs. 17.40%, and MAE: 1.15 vs. 1.14).

Out of the 10 top-ranked predictors of the HAI, 8 predictors are biological exposures. The top 3 encompasses the average BMI, waist circumference, and waist/hip ratio over time, which are all anthropometric measures of overweight and obesity. In addition, 4 cholesterol-related measures are represented in the top 10. The final two predictors are average Dutch Healthy Diet index over time and educational level, which belong to the domains of lifestyle and demographic exposures respectively.

**Fig. 3:**
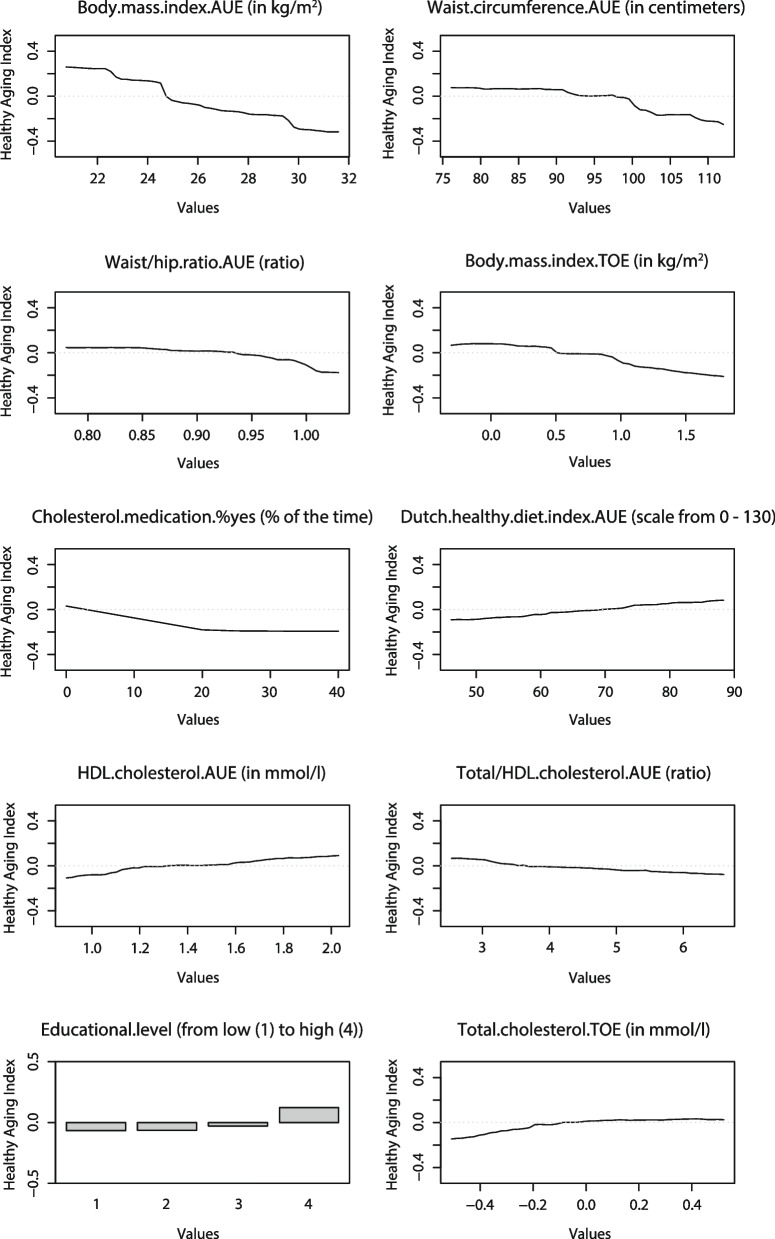
Partial dependence plots (PDPs) of the relation between the 10 top-ranked predictors and the Healthy Aging Index. A positive score indicates a better health. AUE, Area-Under-the-Exposure; TOE, Trend-Of-the-Exposure

The relation between the 10 top-ranked predictors and the HAI was plotted in PDPs (Fig. 3). These plots show that having on average a high BMI, a high waist circumference, and a high waist/hip ratio were predictive of a poorer HAI score. Furthermore, an increase in BMI over time and an increase in percentage of the time using cholesterol lowering medication was also associated with a poorer HAI score. Having a higher score on the Dutch Healthy Diet index 2015 (which indicates higher adherence to the Dutch dietary guidelines) and having a higher educational level was associated with a better HAI score. For cholesterol, having on average a higher HDL cholesterol and a lower total/HDL cholesterol ratio were predictive of a better HAI score. Lastly, a decrease in total cholesterol over time was also predictive of a poorer HAI score, which may be related to medication use among participants with high total cholesterol levels.

### Predictors of healthy physiological aging across generations

All analyzes were repeated for the four different generations separately. For each generation, the parameters of the RF model were tuned, the variable importance ranking was determined, and the number of most important exposures was selected through cross-validation. The quality metrics of the different models are shown in Table S 3. Similar as for the total study population, the prediction performance of the models of the different generations was quite modest. The performance was slightly better for the older generations, e.g. for generation 1960s the RMSE was 1.39 in the null model compared to 1.38 in the final model, while for generation 1930s these numbers were 1.88 and 1.55 respectively.

Table 3 shows the top-ranked predictors of the HAI at wave 6 for the total study population and the four generations separately, and also includes the average ranking list of the top-ranked predictors of all generations together. Among the total study population and all generations, the biological exposures were overrepresented in the top-ranked predictors. The average BMI, waist circumference, and waist/hip ratio over time were important predictors of the HAI for all generations. Moreover, for generation 1960s, 1950s, and 1940s, the average BMI or waist circumference was the single most important predictor, while this was percentage of the time using cholesterol lowering medication for generation 1930s. Similar as for the total population, other important predictors besides anthropometric measures were generally measures related to cholesterol, diet, and educational level. Furthermore, for both generation 1950s and 1940s, a smoking-related measure was found among the top-ranked predictors. No notable differences in the relation between predictors and the HAI across generations was observed in comparison with the PDPs of the total study population.

**Table 3:** Top-ranked predictors of the Healthy Aging Index for the total study population and the four generations, and the average ranking list of the top-ranked predictors of the four generations together

Ranking	Exposures	Domain	Total population (*n* = 2815)	# of times selected as predictor	Average ranking if selected^a^	Generation 1960s (*n* = 459)	Generation 1950s (*n* = 1155)	Generation 1940s (*n* = 872)	Generation 1930s (*n* = 329)
1	Body mass index (AUE)	Biological	1	4	3.0	3	1	1	7
2	Waist/hip ratio (AUE)	Biological	3	4	3.0	4	3	2	3
3	Waist circumference (AUE)	Biological	2	4	3.5	1	2	3	8
4	Body mass index (TOE)	Biological	4	3	6.0	2	5	11	
5	Cholesterol medication (% of the time yes)	Biological	5	2	2.5			4	1
6	Total cholesterol (TOE)	Biological	10	2	5.0			5	5
7	Educational level^b^	Demographic	9	2	6.5		11		2
8	Total/HDL cholesterol (AUE)	Biological	8	2	7.0		4	10	
9	Dutch Healthy Diet index 2015 (AUE)	Lifestyle	6	2	7.5			9	6
10	HDL cholesterol (AUE)	Biological	7	2	7.5		7	8	
11	HDL cholesterol (TOE)	Biological		2	8.0			7	9
12	Total/HDL cholesterol (TOE)	Biological		1	4.0				4
13	Packyears (AUE)	Lifestyle		1	6.0		6		
14	Smoking status (% of the time smoker)	Lifestyle		1	6.0			6	
15	Working hours (AUE)	Demographic		1	8.0		8		
16	PM_2.5_ (AUE)	Environmental		1	9.0		9		
17	Cholesterol medication (% from no to yes)	Biological		1	10.0		10		
18	Waist circumference (TOE)	Biological		1	10.0				10

*AUE* Area-Under-the-Exposure, *TOE* Trend-Of-the-Exposure

The top-ranked predictors were selected through cross-validation (x = 10 for the total population, x = 4 for generation 1960s, x = 11 for generation 1950s, x = 11 for generations 1940s, and x = 10 for generation 1930s)

^a^Calculated by dividing the sum of a particular exposure’s ranking position for all generations by the number of times the exposure has been selected as top-ranked predictor (e.g., for body mass index (AUE), this is (3 + 1 + 1 + 7)/4 = 3.0)

^b^Educational level represents the highest level of education attained and is not a time-varying exposure

## Discussion

Using exposures from multiple domains that were repeatedly measured over 30 years, we created a prediction model for healthy physiological aging with random forest based on data from a 30-year long cohort study. In total, 8 out of 10 of the most important predictors of healthy physiological aging were measures from the biological domain and related to overweight (BMI, waist circumference, waist/hip ratio) and cholesterol (use of cholesterol lowering medication, HDL and total cholesterol). Diet and educational level also ranked in the top of important exposures. No substantial differences were observed in the predictors of healthy physiological aging across generations.

To our knowledge, the current study is the first to predict healthy physiological aging by including longitudinal exposures from multiple domains of the exposome, as opposed to previous mostly cross-sectional risk-assessment studies including only a limited number of exposures [3–5, 7–10]. For this purpose, we used our recently proposed machine learning approach. By doing this, we were able to provide a direct comparison of the importance of the predictors of healthy physiological aging. Our results show that overweight-related and cholesterol-related measures, which are related to the internal exposome, were found most among the top-ranked predictors. In earlier work, overweight has also been identified as an important predictor and determinant of healthy aging [6, 11, 14, 36]. In their review, Tam et al. (2019) explain this by demonstrating that the pathology of overweight and obesity is similar to that of aging, with the same adverse molecular and cellular changes being initiated in obesity and aging [37]. Furthermore, the internal exposome is more directly connected with the health outcome than for example lifestyle and environmental exposures (that are in part determinants of the internal exposome) in the exposome concept [2]. This may therefore explain the relative importance of overweight (i.e. BMI and waist circumference) and cholesterol as *predictors* of healthy physiological aging. In addition, the predictive ability of overweight and cholesterol may be further explained by their known associations with high blood pressure and high blood glucose [38–40], which are both included in the Healthy Aging Index. While associations of overweight and cholesterol with indicators of the HAI were already established in previous epidemiological research [38–40], the current study indicates that these measures are also the most important *predictors* of healthy physiological aging.

Besides biological exposures, educational level and diet were also found to be important predictors of healthy physiological aging. In correspondence, multiple previous studies have identified educational level to be related to healthy aging [6, 8, 10, 11, 14]. This finding may be attributed to the earlier onset of health problems in people with a lower socioeconomic status [41]. With respect to diet, a better dietary quality and higher daily consumption of fruits and vegetables have been found to be associated with a higher probability of healthy aging [3, 4, 7, 42], which may be related to beneficial effects on lipid profiles and the antioxidant capacities of healthy diets [4, 43]. The predictive ability of diet may not only be due to a beneficial association of healthy diet with physical aging, but also due to its association with cognitive aging [44], which are both aspects of the Healthy Aging Index.

Earlier work, including work on the Doetinchem Cohort Study, reported associations between other lifestyle exposures and healthy physiological aging, such as physical activity, smoking, and alcohol use [3, 4, 6, 7, 11, 14, 45]. These exposures were not found to be the most important predictors of healthy physiological aging in the current study, but this does not mean that these lifestyle exposures are not associated with healthy physiological aging. However, an association alone is insufficient to establish predictive value [46]. Our results imply that the biological exposures in combination with educational level and diet are the most important exposures to *predict* healthy physiological aging and addition of other (lifestyle) exposures does not further improve the performance of the prediction model in the current study. Previous studies including multiple lifestyle exposures often used a cumulative score of all lifestyle exposures combined [5, 8, 9]. This limits the possibility to assess the relative contribution of the individual lifestyle exposures in predicting healthy physiological aging.

In the prediction models for the four generations (generation 1960s/1950s/1940s/1930s aged respectively 20–29/30–39/40–49/50–59 years old at baseline) separately, similar predictors of healthy physiological aging were identified as in the total study population. In all models, the average BMI, waist circumference, and waist/hip ratio over time were identified to be among the top-ranked predictors of healthy physiological aging. Unfavorable generation shifts in BMI have previously been observed, with younger generations having a higher BMI than older generations at the same age [12, 47]. The current study indicates that a high BMI is an important predictor of poorer healthy physiological aging across all generations. Since no other studies were found that examined differences in predictors of healthy physiological aging across generations, more research is required to confirm our findings.

In the current study, the performance of the prediction model can be evaluated as being rather modest with a 9% reduction of the RMSE in the final model compared to the null model, and a R^2^ of 17% in the final model. As a large number of exposures from different domains were included, we expected a higher prediction performance. Explanations for the modest discriminative properties of model could be: 1. underestimation of the effect of some exposures, because of measurement error (e.g. in certain exposures that were measured by questionnaire) [48], 2. the possibility that important exposures were not included or not measured in critical periods (e.g. exposures during childhood) [48], and 3. the fact that most lifestyle and environmental exposures generally only have been found to have modest effects on health [49]. The performance of the prediction model was better in the older than in the younger generations (e.g. reduction in RMSE in the final prediction model was 18% among generation 1930s and 1% among generation 1960s, and R^2^ was respectively 32% and 1%). This might indicate that it is more difficult to predict healthy physiological aging earlier in life or that the included exposures were less important predictors of healthy physiological aging earlier in life. As exposures accumulate over the years, the (relative) importance of these exposures may become apparent especially at older ages. In all prediction models, the majority of predictors reflected the average exposure over time (AUE) instead of the average trend in the exposure over time (TOE). This suggests that the *average* exposure level over the life course is more important in predicting healthy physiological aging than the actual *change* in exposure.

A recent systematic review into determinants of healthy aging identified determinants related to physical, social, and mental well-being [50]. In the current study most included exposures were generally related to physical well-being and social well-being. Therefore, including exposures related to mental well-being, such as self-awareness, attitude, and life-long learning [50], could have improved the prediction model of healthy physiological aging. Nevertheless, since most indicators of the Healthy Aging Index are physical indicators and therefore closely related to physical well-being, the relative improvement in the prediction model by adding exposures related to mental well-being may have been limited. Lastly, other measures of socioeconomic status besides education (e.g. wealth and financial security) may be relevant additions to future work aiming to predict healthy physiological aging [6, 50].

### Strengths and limitations

Strengths of the current study are its longitudinal population-based design in which participants were repeatedly measured over the course of 30 years and the inclusion of a wide range of exposures from different domains of the exposome. For these exposures, we studied both the average level of exposure and changes (trend) in exposure over the life course.

The results of the current study apply to the concept of healthy *physiological* aging. However, it should be noted that healthy aging in a broader sense, such as defined by the WHO [1], not only includes physical markers of health, but also the interaction of an individual’s intrinsic capacity with the home, community, and societal environment. Healthy aging is not only about being free of disease and disability, but also about optimal societal participation and mental well-being [50]. For future research, it would therefore be interesting to study whether similar predictors of healthy aging are identified when a broader definition is used. Nevertheless, the HAI used in the current study is an interesting outcome reflecting the physiological aspects of an individual’s intrinsic capacity. The HAI is relatively easy to measure, has shown to be a valid predictor of mortality, morbidity, and disability, and its use as a summary measure of physiological aging has been supported by previous studies [13–17, 51]. By using the HAI, individuals with suboptimal and those with optimal outcomes relevant for healthy aging can be distinguished, resulting in an intermediate endpoint for longevity [51].

A limitation of the current study is that the sample size was relatively small after stratification for the four generations. In particular, a limited number of participants in generation 1960s (*n* = 459) and generation 1930s (*n* = 329) were available. Furthermore, inherent to the longitudinal design of the Doetinchem Cohort Study is the limitation of selective attrition where healthier participants are more likely to remain in the study population over time [19]. In correspondence, participants who were included in the current study were found to be younger, higher educated, and healthier than participants of the Doetinchem Cohort Study who dropped out. This may limit the generalizability of our findings to less healthy populations.

Another limitation is that not all exposures were measured at every measurement wave. For example, diet was only measured from wave 2 through 4. If dietary information would have been available for the wave prior to the measurement of the outcome (i.e. wave 5), this might have further increased the predictive ability of this exposure. Nevertheless, it is important to note that most exposures were measured at every wave. Furthermore, for nearly all exposures in this study, we were able to take into account changes of exposures over the life course by examining both the average exposure over time and the average trend in the exposure over time.

Notably, exposures from the environmental domain were lacking from the top-ranked exposures predicting health. However, uncertainty remains with respect to the predictive value of air pollution, noise, and green space measures in the general Dutch population. This is because the current study was conducted among adults from one particular town in the Netherlands, thereby leading to limited variation in these measures. Therefore, nationwide studies may be needed to determine the importance of physical environment measures. In addition, prediction models should ideally be validated in other study populations to assess their potential to be generalized.

## Conclusion

In this 30-year long cohort study, longitudinal overweight-related exposures were the strongest predictors of healthy physiological aging. This finding was similar across generations. Other important predictors of healthy physiological aging were related to cholesterol levels, diet quality, and educational level. Taken together, our findings suggest that exposures from the biological domain are most important in predicting healthy physiological aging. However, due to the modest performance of the prediction model, more work is needed to better predict healthy physiological aging and to confirm our findings in other study populations. In turn, the resulting insights from these efforts will be valuable in the early identification of people who are likely to age unhealthily.

## Supplementary Information

**Additional file 1.**

## Data Availability

The datasets generated and analyzed during the current study are not publicly available due to ethical restrictions related to participant consent but are available from the corresponding author on reasonable request.

## Abbreviations

AUE: Area-Under-the-Exposure
BMI: Body mass index
DHD15: Dutch Healthy Diet index 2015
HAI: Healthy Aging Index
MAE: Mean-absolute error
MSE: Mean square error
NDVI: Normalized difference vegetation index
PDP: Partial dependence plots
R^2^: Explained variance
RF: Random forest
RMSE: Root mean square error
TOE: Trend-Of-the-Exposure
WHO: World Health Organization
