# Supplementary material for: Predictors of healthy physiological aging across generations in a 30-year population-based cohort study: the Doetinchem Cohort Study
Source: BMC Geriatr. 2023 Feb 23;23:107. doi: 10.1186/s12877-023-03789-2 (PMC9948415; doi:10.1186/s12877-023-03789-2)
Supplement: Supplementary file 1 — Additional file 1. [file 12877_2023_3789_MOESM1_ESM.docx]

**ADDITIONAL FILES**

**Predictors of healthy physiological aging across generations in a 30-year population-based cohort study: the Doetinchem Cohort Study**

Bette Loef, Gerrie-Cor M. Herber, Albert Wong, Nicole A.H. Janssen, Jurriaan Hoekstra, H. Susan J. Picavet, W.M. Monique Verschuren

**Table S1.** Overview of demographic, lifestyle, environmental, and biological exposures included in the current study

| **Exposure** | **Label** | **Wave^1^** |
| --- | --- | --- |
| *Demographic exposures* |  |  |
| Sex | men; women | w1 |
| Educational level (highest level of education attained) | primary education or less; lower vocational education or lower secondary education; intermediate vocational education or higher secondary education; higher vocational education or university | w1-w4 |
| Nationality | Dutch; non-Dutch | w1 |
| Marital status | single, never married; married; widow/widower; divorced | w1-w5 |
| Household composition | with partner; with partner and children; single-parent household; single household; other household | w2-w5 |
| Working hours | in hours per week | w2-w5 |
| *Lifestyle exposures* |  |  |
| Alcohol use | no, never; no, I stopped using alcohol; every now and then, but less than 1 glass per week; yes | w1-w5 |
| Number of glasses of alcohol per day | in glasses per day | w1-w5 |
| Smoking status | smoker; former smoker; never smoker | w1-w5 |
| Number of cigarettes per day | in cigarettes per day | w1-w5 |
| Smoking pack years | in the number of smoking years times the number of packs smoked per day | w1-w5 |
| Occupational physical activity (EPIC Physical Activity Questionnaire (Pols et al. 1997)) | sedentary job; standing job; manual work; heavy manual work; not applicable | w1-w5 |
| Time spent on moderate to vigorous physical activity per week (EPIC Physical Activity Questionnaire (Pols et al. 1997)) | <0.5 hour; 0.5 – 3.5 hours; ≥3.5 hours or more, of which <2 hours vigorous; ≥3.5 hours, of which ≥2 hours or more vigorous | w2-w5 |
| Dutch Healthy Diet index 2015 (Looman et al. 2017) | on a scale from 0 – 130 (a higher score indicates higher adherence to the Dutch dietary guidelines) | w2-w4 |
| Number of hours of sleep per day | ≤5 hours; 6 hours; 7 hours; 8 hours; ≥9 hours | w1-w5 |
| Reproductive cycle status | men; women, regular cycle; women, irregular cycle; women, pregnant; women, anticontraceptive or hormone use; women, unknown/surgery; women, menopause | w1-w5 |
| *Environmental exposures* |  |  |
| Total NO_2_ concentration at home address (dispersion models (Velders et al. 2020))^2^ | in ug/m^3^ | w1-w5 |
| Total PM_2.5_ concentration at home address (dispersion models (Velders et al. 2020))^2^ | in ug/m^3^ | w1-w5 |
| Total elemental carbon concentration at home address (dispersion models (Velders et al. 2020))^2^ | in ug/m^3^ | w1-w5 |
| Rail traffic noise levels in 2016 for the entire 24-hour period at home address (Standard Model Instrumentation for Noise Assessments (Schreurs et al. 2010)) | in dB | w1-w5 |
| Road traffic noise levels in 2016 for the entire 24-hour period at home address (Standard Model Instrumentation for Noise Assessments (Schreurs et al. 2010)) | in dB | w1-w5 |
| Normalized difference vegetation index in 2010 in buffer 300 meters around home address (Landsat 5 Thematic Mapper (United States Geological Service) | on a scale from 0 – 1 (higher score indicating more greenness) | w1-w5 |
| Normalized difference vegetation index in 2010 in buffer 1000 meters around home address (Landsat 5 Thematic Mapper (United States Geological Service)) | on a scale from 0 – 1 (higher score indicating more greenness) | w1-w5 |
| **Exposure** | **Label** | **Wave^1^** |
| Damp stains in the house in the past two years | not at all; occasionally; often; always | w2-w3 |
| Mold growth in the house in the past two years | not at all; occasionally; often; always | w2-w3 |
| Hot water supply in the house | geyser with drain; geyser without drain; boiler; combi boiler; combination or other | w2-w3 |
| Heat source for cooking | gas; electric; combination or other | w2-w3 |
| Pet (cat, dog, bird or rodent) in the house | yes; no, not anymore; no, never | w2-w3 |
| Smoking in the participant's environment | yes, at home and at work; yes, at home; yes, at work; no | w2-w3 |
| Social support measured by positive social experiences (Van Oostrom et al. 1995) | on a scale from 8 – 32 (higher score indicates more positive experiences) | w1-w3 |
| Social support measured by negative social experiences (Van Oostrom et al. 1995) | on a scale from 8 – 32 (higher score indicates more negative experiences) | w1-w3 |
| Social support measure for elderly (Van Eijk et al. 1994) | on a scale from 12 – 48 (higher score indicates more social support) | w5 |
| Loneliness scale (De Jong-Gierveld et al. 1985) | on a scale from 0 – 11 (higher score indicates more loneliness) | w5 |
| *Biological exposures* |  |  |
| Body mass index | in kg/m^2^ | w1-w5 |
| Waist/hip ratio | ratio | w2-w5 |
| Waist circumference | in centimeters | w2-w5 |
| Total cholesterol | in mmol/l | w1-w5 |
| HDL cholesterol | in mmol/l | w1-w5 |
| Total/HDL cholesterol ratio | ratio | w1-w5 |
| Use of cholesterol lowering medication | yes; no | w1-w5 |

^1^ Measurement waves during which an exposure was measured (wave 1 20-59 years, wave 2 26-65 years, wave 3 31-70 years, wave 4 36-75 years, wave 5 41-80 years).

^2^ Based on concentration estimates of the year 2000 for wave 1-3; the average of the years 2000 and 2010 for wave 4; and the year 2010 for wave 5.

**Text S1. Longitudinal exposures**

All exposures that were measured during multiple waves of the study were summarized in the average of the exposure at wave 1 through 5 (called the Area-Under-the-Exposure, AUE) and the average trend in the exposure over time (called the Trend-of-the-Exposure, TOE). The AUE is computed by plotting observed exposure values against waves, connecting the values with lines, and determining the average area under these lines (continuous exposures) or by calculating the proportion of waves that the individual occupied a certain state (categorical exposures). The TOE is computed through determining the slope in exposure for each pair of subsequent waves, and taking the average over that (for continuous exposures) or through determining whether a change from one reference category to another category occurred during the waves (categorical exposures).

An advantage of using the AUE and TOE is that they can also be calculated in case of missing values. To calculate the longitudinal exposures, participants had to have a value for the exposure in at least two rounds. Otherwise, that particular longitudinal exposure of the participant was labelled as missing (i.e. 99999, this is an outlier value for continuous exposures and a missing category for categorical exposures). In total, 76% of the participants had no missing values on the longitudinal exposures, 7% of the participants had a missing value on one longitudinal exposure, 5% on two longitudinal exposures, and 12% on three or more longitudinal exposures.

**Table S2.** Characteristics of the study population

| **Exposure/outcome** | **Type** | **Label** | **Total (n=2815)** | | **Generation 1960s (n=459)** | | **Generation 1950s(n=1155)** | | **Generation 1940s (n=872)** | | **Generation 1930s (n=329)** | |
| --- | --- | --- | --- | --- | --- | --- | --- | --- | --- | --- | --- | --- |
|  |  |  | *Mean/%* | *SD/n* | *Mean/%* | *SD/n* | *Mean/%* | *SD/n* | *Mean/%* | *SD/n* | *Mean/%* | *SD/n* |
| *Demographic exposures* |  |  |  |  |  |  |  |  |  |  |  |  |
| Sex | % | women | 53.3 | 1501 | 59.3 | 272 | 52.5 | 606 | 53.0 | 462 | 48.9 | 161 |
| Age^1^ | AUE | in years | 49.4 | 8.4 | 37.9 | 1.7 | 45.6 | 3.0 | 54.7 | 2.8 | 64.5 | 2.7 |
| Age^1^ | TOE | in years | 5.2 | 0.1 | 5.2 | 0.1 | 5.2 | 0.1 | 5.2 | 0.1 | 5.2 | 0.1 |
| Educational level | % | higher vocational education or university | 26.7 | 752 | 22.4 | 103 | 30.9 | 357 | 24.2 | 211 | 24.6 | 81 |
| Nationality | % | Dutch | 99.3 | 2795 | 98.9 | 454 | 99.4 | 1148 | 99.3 | 866 | 99.4 | 327 |
| Marital status | % of the time | married | 82.9 | 31.4 | 73.2 | 33.9 | 85.6 | 28.9 | 86.2 | 30.5 | 78.3 | 35.5 |
| Marital status | % of the time | widow/widower | 2.9 | 13.2 | 0.7 | 6.0 | 0.9 | 6.6 | 2.9 | 12.8 | 12.9 | 27.1 |
| Marital status | % of the time | divorced | 5.8 | 18.9 | 4.4 | 13.1 | 5.6 | 17.1 | 7.7 | 23.8 | 3.6 | 16.9 |
| Marital status | % | from married to widowed or divorced | 16.4 | 462 | 16.2 | 74 | 15.0 | 173 | 15.0 | 131 | 25.5 | 84 |
| Household composition | % of the time | with partner (and children) | 84.9 | 29.7 | 83.6 | 29.0 | 87.0 | 26.5 | 85.8 | 30.5 | 77.2 | 37.2 |
| Household composition | % of the time | single-parent household | 4.1 | 13.5 | 7.1 | 17.5 | 5.3 | 14.8 | 2.1 | 9.5 | 1.3 | 9.6 |
| Household composition | % of the time | single household | 9.2 | 24.7 | 6.6 | 20.8 | 5.9 | 19.1 | 10.8 | 26.7 | 20.1 | 35.6 |
| Household composition | % | from with partner (and children) to single-parent or single household | 13.9 | 386 | 19.5 | 88 | 15.2 | 173 | 9.4 | 82 | 13.2 | 43 |
| Working hours | AUE | in hours per week | 20.9 | 15.8 | 27.0 | 14.7 | 26.4 | 14.7 | 17.2 | 14.2 | 3.5 | 6.4 |
| Working hours | TOE | in hours per week | -1.2 | 7.5 | 2.2 | 6.4 | 1.1 | 6.8 | -5.1 | 7.4 | -3.2 | 6.0 |
| *Lifestyle exposures* |  |  |  |  |  |  |  |  |  |  |  |  |
| Alcohol use | % of the time | no, I stopped using alcohol | 1.6 | 8.1 | 1.4 | 6.4 | 1.4 | 7.8 | 1.7 | 8.6 | 2.4 | 9.3 |
| Alcohol use | % of the time | every now and then or yes | 90.3 | 24.5 | 86.6 | 28.6 | 92.6 | 20.6 | 90.7 | 24.4 | 85.8 | 29.4 |
| Alcohol use | % | from never user to current user | 8.0 | 225 | 11.6 | 53 | 8.1 | 93 | 6.1 | 53 | 7.9 | 26 |
| Number of glasses of alcohol | AUE | in glasses per day | 1.1 | 1.2 | 0.8 | 1.1 | 1.1 | 1.2 | 1.2 | 1.3 | 1.0 | 1.1 |
| Number of glasses of alcohol | TOE | in glasses per day | 0.0 | 0.3 | 0.0 | 0.3 | 0.0 | 0.3 | 0.0 | 0.3 | 0.0 | 0.3 |
| Smoking status | % of the time | smoker | 22.0 | 35.2 | 27.4 | 37.6 | 24.4 | 37.0 | 20.0 | 33.7 | 11.3 | 26.0 |
| Smoking status | % of the time | former smoker | 41.2 | 42.3 | 28.4 | 37.4 | 41.7 | 42.4 | 43.8 | 42.5 | 50.3 | 44.4 |
| Smoking status | % | from never smoker to smoker | 1.6 | 44 | 2.4 | 11 | 1.1 | 13 | 1.8 | 16 | 1.2 | 4 |
| Number of cigarettes | AUE | in cigarettes per day | 13.5 | 6.7 | 12.6 | 5.7 | 14.5 | 6.8 | 12.8 | 7.2 | 12.5 | 6.1 |
| Number of cigarettes | TOE | in cigarettes per day | -0.2 | 3.4 | -0.2 | 2.9 | -0.1 | 3.8 | -0.3 | 3.1 | -0.6 | 2.8 |
| Smoking pack years | AUE | in pack years | 8.8 | 11.5 | 5.2 | 7.0 | 8.4 | 10.3 | 9.9 | 12.8 | 12.0 | 15.2 |
| **Exposure/outcome** | **Type** | **Label** | **Total (n=2815)** | | **Generation 1960s (n=459)** | | **Generation 1950s(n=1155)** | | **Generation 1940s (n=872)** | | **Generation 1930s (n=329)** | |
|  |  |  | *Mean/%* | *SD/n* | *Mean/%* | *SD/n* | *Mean/%* | *SD/n* | *Mean/%* | *SD/n* | *Mean/%* | *SD/n* |
| Smoking pack years | TOE | in pack years | 0.8 | 2.2 | 0.9 | 1.7 | 1.0 | 2.2 | 0.6 | 2.1 | 0.6 | 2.5 |
| Occupational physical activity | % of the time | sedentary job | 28.4 | 35.6 | 33.8 | 37.9 | 35.9 | 39.0 | 23.0 | 31.2 | 9.1 | 15.9 |
| Occupational physical activity | % of the time | standing job | 22.8 | 26.8 | 26.1 | 29.6 | 24.2 | 28.8 | 22.3 | 25.4 | 14.5 | 14.6 |
| Occupational physical activity | % of the time | (heavy) manual work | 21.6 | 30.8 | 28.4 | 34.4 | 26.1 | 33.5 | 17.2 | 26.7 | 8.1 | 16.2 |
| Occupational physical activity | % | from sedentary/standing job to (heavy) manual work | 15.7 | 441 | 22.1 | 101 | 20.3 | 234 | 10.8 | 94 | 3.7 | 12 |
| Moderate to vigorous physical activity | % of the time | <3.5 hours per week | 19.7 | 28.8 | 23.2 | 30.8 | 19.0 | 28.4 | 19.1 | 28.2 | 19.0 | 28.7 |
| Moderate to vigorous physical activity | % | from ≥3.5 hours to <3.5 hours per week | 32.8 | 915 | 36.5 | 165 | 31.9 | 363 | 29.7 | 258 | 39.4 | 129 |
| Dutch Healthy Diet index 2015 | AUE | on a scale from 0 – 130 | 66.1 | 11.8 | 63.0 | 11.2 | 65.7 | 11.7 | 67.2 | 11.8 | 69.3 | 12.0 |
| Dutch Healthy Diet index 2015 | TOE | on a scale from 0 – 130 | 1.2 | 6.5 | 0.8 | 6.7 | 1.0 | 6.4 | 1.3 | 6.4 | 1.6 | 6.4 |
| Number of hours of sleep | AUE | in hours per day (1=≤5, 2=6, 3=7, 4=8, 5=≥9 hours) | 3.3 | 0.7 | 3.3 | 0.6 | 3.2 | 0.7 | 3.3 | 0.7 | 3.4 | 0.8 |
| Number of hours of sleep | TOE | in hours per day (1=≤5, 2=6, 3=7, 4=8, 5=≥9 hours) | -0.1 | 0.3 | -0.2 | 0.3 | -0.1 | 0.2 | -0.1 | 0.3 | 0.0 | 0.3 |
| Reproductive cycle status | % of the time | women, regular cycle | 20.8 | 28.7 | 37.9 | 37.8 | 25.0 | 29.7 | 13.6 | 19.3 | 1.6 | 6.3 |
| Reproductive cycle status | % of the time | women, irregular cycle | 4.5 | 11.1 | 6.1 | 13.0 | 5.7 | 12.6 | 3.2 | 8.7 | 1.6 | 5.8 |
| Reproductive cycle status | % of the time | women, menopause | 17.2 | 27.7 | 1.6 | 6.2 | 11.5 | 16.9 | 25.5 | 30.8 | 37.1 | 44.5 |
| Reproductive cycle status | % | from regular cycle to irregular cycle or menopause | 38.0 | 1070 | 44.6 | 204 | 45.2 | 522 | 36.9 | 322 | 6.7 | 22 |
| *Environmental exposures* |  |  |  |  |  |  |  |  |  |  |  |  |
| NO_2_ concentration | AUE | in ug/m^3^ | 27.7 | 1.8 | 27.5 | 1.7 | 27.8 | 1.7 | 27.7 | 2.0 | 28.0 | 2.1 |
| NO_2_ concentration | TOE | in ug/m^3^ | -1.6 | 0.6 | -1.6 | 0.7 | -1.6 | 0.6 | -1.6 | 0.6 | -1.5 | 0.5 |
| PM_2.5_ concentration | AUE | in ug/m^3^ | 20.2 | 0.6 | 20.2 | 0.5 | 20.2 | 0.6 | 20.2 | 0.6 | 20.3 | 0.6 |
| PM_2.5_ concentration | TOE | in ug/m^3^ | -1.8 | 0.4 | -1.8 | 0.4 | -1.8 | 0.4 | -1.8 | 0.4 | -1.7 | 0.4 |
| Elemental carbon concentration | AUE | in ug/m^3^ | 1.3 | 0.1 | 1.3 | 0.1 | 1.3 | 0.1 | 1.3 | 0.1 | 1.3 | 0.2 |
| Elemental carbon concentration | TOE | in ug/m^3^ | -0.1 | 0.0 | -0.1 | 0.1 | -0.1 | 0.0 | -0.1 | 0.0 | -0.1 | 0.0 |
| Rail traffic noise levels | AUE | in dB | 31.8 | 7.4 | 31.4 | 6.5 | 32.4 | 7.3 | 31.3 | 7.6 | 31.8 | 8.1 |
| Rail traffic noise levels^2^ | TOE | in dB | 0.0 | 1.9 | 0.2 | 2.0 | 0.0 | 2.0 | -0.1 | 1.7 | 0.1 | 1.5 |
| Road traffic noise levels | AUE | in dB | 52.5 | 5.1 | 52.4 | 4.7 | 52.2 | 5.0 | 52.6 | 5.1 | 53.6 | 5.9 |
| Road traffic noise levels^2^ | TOE | in dB | -0.1 | 1.4 | -0.2 | 1.6 | -0.1 | 1.5 | -0.1 | 1.4 | 0.1 | 1.2 |
| NDVI in 300 meters buffer | AUE | on a scale from 0 – 1 | 0.5 | 0.1 | 0.5 | 0.1 | 0.5 | 0.1 | 0.5 | 0.1 | 0.5 | 0.1 |
| **Exposure/outcome** | **Type** | **Label** | **Total (n=2815)** | | **Generation 1960s (n=459)** | | **Generation 1950s(n=1155)** | | **Generation 1940s (n=872)** | | **Generation 1930s (n=329)** | |
|  |  |  | *Mean/%* | *SD/n* | *Mean/%* | *SD/n* | *Mean/%* | *SD/n* | *Mean/%* | *SD/n* | *Mean/%* | *SD/n* |
| NDVI in 300 meters buffer^2^ | TOE | on a scale from 0 – 1 | 0.0 | 0.0 | 0.0 | 0.0 | 0.0 | 0.0 | 0.0 | 0.0 | 0.0 | 0.0 |
| NDVI in 1000 meters buffer | AUE | on a scale from 0 – 1 | 0.5 | 0.1 | 0.5 | 0.1 | 0.5 | 0.1 | 0.5 | 0.1 | 0.5 | 0.1 |
| NDVI in 1000 meters buffer^2^ | TOE | on a scale from 0 – 1 | 0.0 | 0.0 | 0.0 | 0.0 | 0.0 | 0.0 | 0.0 | 0.0 | 0.0 | 0.0 |
| Damp stains in the house | % of the time | occasionally or often or always | 22.1 | 34.3 | 25.9 | 35.2 | 23.6 | 34.8 | 20.7 | 34.2 | 15.5 | 30.6 |
| Damp stains in the house | % | from not at all to occasionally or often or always | 9.4 | 238 | 12.2 | 49 | 10.4 | 106 | 8.4 | 67 | 5.1 | 16 |
| Mold growth in the house | % of the time | occasionally or often or always | 11.4 | 25.8 | 12.5 | 25.4 | 14.0 | 28.2 | 9.4 | 23.9 | 6.4 | 21.0 |
| Mold growth in the house | % | from not at all to occasionally or often or always | 7.6 | 193 | 11.8 | 47 | 9.4 | 96 | 5.5 | 44 | 1.9 | 6 |
| Hot water supply in the house | % of the time | combi boiler | 51.8 | 42.7 | 57.4 | 40.2 | 50.7 | 43.4 | 51.8 | 42.0 | 48.7 | 44.8 |
| Hot water supply in the house | % of the time | combination or other | 16.9 | 30.0 | 17.7 | 29.3 | 18.3 | 31.7 | 16.0 | 28.7 | 13.9 | 28.4 |
| Hot water supply in the house | % | from geyser to combi boiler | 8.8 | 224 | 10.0 | 40 | 8.3 | 85 | 9.6 | 77 | 7.0 | 22 |
| Heat source for cooking | % of the time | gas | 68.6 | 42.8 | 70.9 | 40.1 | 71.7 | 41.6 | 63.4 | 44.9 | 69.1 | 43.4 |
| Heat source for cooking | % | from gas to electric | 6.7 | 170 | 10.4 | 42 | 6.0 | 61 | 6.2 | 50 | 5.4 | 17 |
| Pet in the house | % of the time | yes | 48.9 | 44.5 | 50.9 | 43.5 | 54.5 | 43.9 | 47.3 | 45.0 | 32.2 | 42.7 |
| Pet in the house | % | from yes to no | 12.2 | 311 | 12.0 | 48 | 11.9 | 122 | 13.2 | 106 | 11.1 | 35 |
| Smoking in participant's environment | % of the time | yes, at home and/or at work | 45.2 | 44.4 | 49.0 | 43.8 | 47.7 | 44.8 | 45.5 | 44.6 | 31.7 | 40.6 |
| Smoking in participant's environment | % | from no to yes | 6.0 | 153 | 7.0 | 28 | 7.2 | 74 | 4.7 | 38 | 4.1 | 13 |
| Social support (positive experiences) | AUE | on a scale from 8 – 32 | 22.9 | 3.1 | 23.4 | 3.0 | 23.0 | 3.1 | 22.6 | 3.2 | 22.6 | 2.9 |
| Social support (positive experiences) | TOE | on a scale from 8 – 32 | 0.4 | 2.1 | 0.2 | 2.2 | 0.5 | 2.1 | 0.4 | 2.1 | 0.5 | 2.1 |
| Social support (negative experiences) | AUE | on a scale from 8 – 32 | 12.7 | 2.4 | 12.7 | 2.3 | 12.7 | 2.3 | 12.8 | 2.4 | 12.1 | 2.3 |
| Social support (negative experiences) | TOE | on a scale from 8 – 32 | 0.2 | 1.7 | 0.4 | 1.7 | 0.4 | 1.7 | 0.2 | 1.8 | -0.3 | 1.6 |
| Social support measure for elderly | mean w5 | on a scale from 12 – 48 | 30.5 | 5.6 | 30.7 | 5.3 | 30.6 | 5.6 | 30.4 | 5.6 | 29.6 | 5.7 |
| Loneliness scale | mean w5 | on a scale from 0 – 11 | 2.1 | 2.7 | 1.8 | 2.5 | 2.0 | 2.7 | 2.2 | 2.6 | 2.6 | 2.8 |
| *Biological exposures* |  |  |  |  |  |  |  |  |  |  |  |  |
| Body mass index | AUE | in kg/m^2^ | 25.6 | 3.5 | 24.6 | 3.3 | 25.4 | 3.4 | 26.0 | 3.4 | 26.6 | 3.5 |
| Body mass index | TOE | in kg/m^2^ | 0.6 | 0.7 | 0.8 | 0.8 | 0.7 | 0.7 | 0.5 | 0.7 | 0.4 | 0.6 |
| Waist/hip ratio | AUE | ratio | 0.9 | 0.1 | 0.9 | 0.1 | 0.9 | 0.1 | 0.9 | 0.1 | 0.9 | 0.1 |
| Waist/hip ratio | TOE | ratio | 0.0 | 0.0 | 0.0 | 0.0 | 0.0 | 0.0 | 0.0 | 0.0 | 0.0 | 0.0 |
| Waist circumference | AUE | in centimeters | 92.6 | 10.6 | 88.7 | 10.4 | 91.9 | 10.4 | 94.2 | 10.4 | 96.6 | 10.1 |
| Waist circumference | TOE | in centimeters | 2.1 | 2.6 | 2.2 | 2.9 | 2.3 | 2.6 | 1.9 | 2.4 | 1.7 | 2.3 |
| Total cholesterol | AUE | in mmol/l | 5.5 | 0.8 | 5.1 | 0.8 | 5.4 | 0.8 | 5.7 | 0.8 | 5.8 | 0.8 |
| **Exposure/outcome** | **Type** | **Label** | **Total (n=2815)** | | **Generation 1960s (n=459)** | | **Generation 1950s(n=1155)** | | **Generation 1940s (n=872)** | | **Generation 1930s (n=329)** | |
|  |  |  | *Mean/%* | *SD/n* | *Mean/%* | *SD/n* | *Mean/%* | *SD/n* | *Mean/%* | *SD/n* | *Mean/%* | *SD/n* |
| Total cholesterol | TOE | in mmol/l | 0.1 | 0.3 | 0.1 | 0.2 | 0.1 | 0.3 | 0.0 | 0.3 | -0.1 | 0.3 |
| HDL cholesterol | AUE | in mmol/l | 1.4 | 0.4 | 1.4 | 0.3 | 1.4 | 0.4 | 1.4 | 0.4 | 1.4 | 0.3 |
| HDL cholesterol | TOE | in mmol/l | 0.0 | 0.1 | 0.0 | 0.1 | 0.0 | 0.1 | 0.0 | 0.1 | 0.0 | 0.1 |
| Total/HDL cholesterol ratio | AUE | ratio | 4.2 | 1.3 | 3.9 | 1.2 | 4.2 | 1.3 | 4.3 | 1.3 | 4.5 | 1.1 |
| Total/HDL cholesterol ratio | TOE | ratio | -0.1 | 0.4 | 0.0 | 0.3 | 0.0 | 0.4 | -0.1 | 0.4 | -0.3 | 0.4 |
| Use of cholesterol lowering medication | % of the time | yes | 5.3 | 14.2 | 1.0 | 5.5 | 3.3 | 10.8 | 7.4 | 16.0 | 12.9 | 21.9 |
| Use of cholesterol lowering medication | % | from no to yes | 12.2 | 342 | 2.8 | 13 | 9.4 | 108 | 16.7 | 146 | 22.8 | 75 |
| *Outcome* |  |  |  |  |  |  |  |  |  |  |  |  |
| Healthy Aging Index (crude) | at wave 6 | on a scale from 0 – 10 | 7.2 | 1.8 | 8.2 | 1.3 | 7.7 | 1.5 | 6.7 | 1.8 | 5.5 | 1.8 |
| Age-adjusted Healthy Aging Index | at wave 6 | on a scale from -10 – 10 | 0.0 | 1.6 | 0.0 | 1.3 | 0.0 | 1.5 | 0.0 | 1.7 | 0.0 | 1.8 |

^1^ Age is shown here for descriptive purposes only, as age is not included as an exposure in the analyses, and only used to create the age-adjusted Healthy Aging Index outcome.

^2^ The noise and NDVI estimates are based on the year 2016 and 2010, respectively. The TOE values for these estimates only change when participants change home addresses.

AUE, Area-Under-the-Exposure; NDVI, Normalized difference vegetation index; TOE, Trend-Of-the-Exposure.

**Table S3.** Quality metrics of the null model^1^ and the final random forest model^2^ predicting the Healthy Aging Index for the total study population and the four generations separately.

|  | **Total population (n=2815)** | | **Generation 1960s (n=459)** | | **Generation 1950s (n=1155)** | | **Generation 1940s (n=872)** | | **Generation 1930s (n=329)** | |
| --- | --- | --- | --- | --- | --- | --- | --- | --- | --- | --- |
|  | *Null model* | *Final model* | *Null model* | *Final model* | *Null model* | *Final model* | *Null model* | *Final model* | *Null model* | *Final model* |
| Exposures | 0 | 10 | 0 | 4 | 0 | 11 | 0 | 11 | 0 | 10 |
| RMSE | 1.58 | 1.44 | 1.39 | 1.38 | 1.35 | 1.23 | 1.78 | 1.60 | 1.88 | 1.55 |
| R^2^ | 0.00 | 17.00 | 0.01 | 0.93 | 0.00 | 17.13 | 0.00 | 18.44 | 0.00 | 31.88 |
| MAE | 1.27 | 1.15 | 1.11 | 1.05 | 1.11 | 0.98 | 1.41 | 1.25 | 1.53 | 1.22 |

^1^ Null model: metrics of the model without any exposures that predicts the training dataset mean.

^2^ Final model: metrics of random forest model fitted on test dataset with optimal parameter settings including a x number of top-ranked exposures.

MAE, mean-absolute error; R^2^, explained variance; RMSE, the root mean square error.
